# Some Remarks on Necrosis, Illustrated by a Case

**Published:** 1821-10

**Authors:** A. Copland Hutchison

**Affiliations:** Senior Surgeon to the Westminster General Dispensary, and to the Royal Metropolitan Infirmary for Sick Children, &c.


*Some Remarks on Necrosis, illustrated by a Case.* By A. Copland Hutchison, Senior Surgeon to the Westminster General Dispensary, and to the Royal Metropolitan Infirmary for Sick Children, &c.

SEVERAL cases of necrosed tibia have occurred to me, at various periods, and some of them, apparently, the result of attacks of erysipelas, either spontaneous or produced by blows or bruises on the shin-bone. It is my intention at present, therefore, to detail the particular circumstances of one case only, because of most recent date, attended with the most remarkable kind of sequestra, and because the treatment pursued has, in all, been the same.

In stating that some of the cases adverted to appeared to be the sequelæ of erysipelatous inflammation, I should wish it to be understood that not any of the patients were placed under my care until after the disease termed necrosis was completely developed; and that I have never known an instance of necrosis resulting from such species of inflammation, when the plan of treatment recommended by me, in erysipelas, had been pursued.* I may take this opportunity to mention, also, that in the few cases of erysipelas admitted at the Westminster General Dispensary within the last five years, and treated by incision; every individual case terminated favourably: some of my colleagues and pupils can bear testimony to the fact.

To prove that erysipelas, and new depositions of bony matter, are sometimes the consequence of blows, bruises, and lacerations of the periosteum, we have only to instance, in the first place, the frequent attacks of this species of inflammation on the face and leg, after such accidents to the head and tibia: and, to prove the latter, we need but advert to the cases daily

* See some Practical Observations in Surgery, published in 1816.

witnessed in practice, of the cut end of the femur, after amputation, frequently ending in necrosis, owing to sufficient attention not having been paid to saw the bone through exactly where the periosteum is divided, but higher up, or above the part, so as to tear and lacerate this membrane with the teeth of the saw; and the recorded consequences of passing a seton through the ununited parts of a fractured bone, prove both facts.

I know not that I should, at present, have been induced to lay these remarks and the subjoined case before the public, were it not to fulfil a promise made to Mr. Russell, the distinguished professor of clinical surgery in the university of Edinburgh, whom I accidently met last week at the house of a mutual medical friend.

John West, aged 26, a shipwright in Woolwich Dock-yard, was, in the year 1814, attacked with erysipelatous inflammation of the right leg, extending from the knee to the ankle: this disease, after some time, terminated in two or three small openings, from which a discharge of ill-conditioned pus issued. The erysipelatous redness of the skin disappeared with the formation of these little abscesses which ended in the openings already mentioned; but the accompanying swelling, or rather enlargement of the limb, continued. The patient, who was confined to the house during the period of acute inflammation, was now enabled to return to his duty in the dock-yard, and continued at his occupation until November 1816; when his rest at night was very much broken by the pain of his leg, and his appetite had become much impaired.

About this period the patient consulted me, when there were several openings in the course of the tibia, amounting, I think, to seven; and, on introducing a probe into some of these small apertures, the instrument descended an inch and upwards, in a sort of narrow bony case. The size of the tibia was thrice that of its natural dimensions. The discharge through these little openings was considerable, thin, bloody, and of a disagreeable smell. His pulse was hectic, and his countenance sallow.

In this state of the case I proposed to him an operation to remove the sequestra, there being no doubt of the nature of the disease. To this proposal the patient readily consented, as he had prepared his mind for nothing short of amputation of the limb above the knee. Accordingly, in December 1816, in the presence of my friends, Mr. Brookes, of Blenheim-street, Drs. Dunn and Tainsh, of Woolwich, and Mr. Coomb, of the same place, I made an incision on the fore part of the tibia, ten inches in length, commencing at the tuberosity of the tibia, and carrying it downwards in the direction of the ulcerated openings. The integuments and periosteum, on each side of this extensive incision, were dissected off from the enlarged bone; and the crown of a full-sized trephine was applied immediately over the lower opening or ulceration, about three inches above the articular end of the bone; but, after cutting down to the shoulder of the instrument, a full inch in depth, the circular piece, so cut by the saw, could not be detached. The trephine was now applied immediately above the last perforation, when a circular portion was cut out, of three-fourths of an inch in thickness, of solid compact bone, with a fine membrane, or periosteum, lining its inner surface. On the introduction of a finger into this aperture made by the trephine, the rough sequestra was distinctly felt at the bottom, but apparently immoveable. Similar portions were successively cut out upwards, to the number of seven in all, each portion being of the same thickness, density, or compactness of bone, and having the same smooth membrane, or periosteum, lining its under surface.

This continuous line of perforations in the new osseous shell was made a straight perpendicular excavation, by cutting, with Hey's head-saw, through the angular pieces left by the trephine, down to the dead bone, or sequestra.

The first circular portion cut by the trephine was removed by means of a chissel and mallet, as recommended by Mr. Russel in such cases.* The portion of dead bone lying at the bottom of this great bony cavity was now found to move a little upwards and downwards, when given that motion to by the application of the finger, but could not be detached whole without extending the osseous cavity on each side at its fundus, by means of the aforesaid chissel and mallet; when, to our surprise, we found the sequestra nothing more than an exfoliated lamina of bone, lying upon a healthy granulated surface of the old tibia.

The whole operation occupied upwards of three hours, of the hardest labour I had ever before experienced; and, when completed, the limb had a frightful appearance to all around. There was very little blood lost; nor did the patient appear to suffer so much as one would have expected, excepting when the periosteum was dissecting off.

The patient was prescribed an anodyne, and left under the good care and management of Dr. Dunn, surgeon of the dock-yard; and, some months after the operation, he walked to London from Woolwich, to show his leg, and to thank me for what had been done.

I saw West some time during the last year, when there still continued a slight discharge from a small opening in the line of

---

* See Russel on Necrosis, published in 1794.

the excavation, but which had not precluded him from following his avocations during the preceding three years; and the immense enlargement of the limb had become so much reduced by the gradual absorption of the remaining new-formed bone, that it seemed a very little larger only than the sound one.

The operation described in the above case I have performed six times, and succeeded in restoring the limbs of all of the patients, excepting one, who, during the cure, had his wound attacked with hospital gangrene, and subsequently died at Deal Hospital, after having suffered amputation above the knee.

Some of the portions of bone removed in the operation described, are in the museum of Mr. Brooks.

*Leicester-square; August* 31*st*, 1821.

P. S.—The author went to Woolwich since the above was written, and found that West had, up to this period, continued, without interruption, at his daily labour; and that his leg exhibited, pretty nearly, the same appearances as last year, there still remaining some enlargement and a slight discharge, but no other inconvenience.

